# Bovine Uterine Microbiota and Endometritis: Ecological Characteristics, Host Interactions, Inflammatory Regulation, and Control Strategies in Dairy Cows

**DOI:** 10.3390/ani16121860

**Published:** 2026-06-16

**Authors:** Yongqi Liu, Shuaiyu Wang

**Affiliations:** College of Veterinary Medicine, China Agricultural University, No. 2 Yuanmingyuan West Road, Haidian District, Beijing 100193, China; s20243051149@cau.edu.cn

**Keywords:** dairy cow, endometritis, uterine microbiota, postpartum disease, dysbiosis, innate immunity, inflammatory persistence

## Abstract

Bovine endometritis remains one of the most significant postpartum uterine disorders. It impairs uterine recovery, compromises fertility, and causes substantial economic losses in dairy production. Growing evidence suggests that the disease cannot be attributed solely to postpartum bacterial contamination; rather, it should be understood as a multifactorial failure to restore uterine homeostasis after calving. This review summarises the latest research findings on six interconnected aspects: the clinical significance of postpartum uterine disease; the diagnostic and biological differences between clinical and subclinical endometritis; the role of microbes in the uterus in health and disease; interactions between the host and uterine bacteria; the mechanisms of persistent inflammatory regulation; and current as well as emerging treatment strategies. Current evidence indicates that postpartum uterine disease is more strongly associated with dysbiosis, reduced microbial diversity, and disturbed microbial succession than with the presence of any single pathogen. Disease progression is driven by complex interplay among microbial ligands, epithelial and stromal immune responses, virulence-associated tissue injury, endocrine disruption, and impaired inflammatory resolution. Furthermore, persistent uterine inflammation is regulated by multilayered networks involving cytokines, prostaglandins, noncoding RNAs, extracellular vesicles, metabolic remodeling, and oxidative stress. Although conventional therapies remain relevant in certain clinical cases, microbiota-oriented approaches, particularly probiotic interventions, have emerged as promising adjunctive strategies for the prevention and control of the condition. Overall, bovine endometritis should be viewed as a disorder caused by disrupted interactions between the host, microbiota and inflammation. Future progress will depend on longitudinal, strain-resolved, and function-oriented studies to enable more precise and less antimicrobial-dependent interventions for postpartum uterine health.

## Simple Summary

Endometritis is a common uterine disease in dairy cows after calving and is a major cause of poor fertility and economic loss on farms. Traditionally, it was mainly regarded as a bacterial infection. However, recent studies show that the disease is better understood as a failure of the uterus to restore normal balance after calving. This review explains that uterine disease is linked not only to the presence of bacteria but also to changes in microbial diversity, disrupted microbial succession, abnormal immune responses, tissue damage, and prolonged inflammation. It also highlights that clinical and subclinical endometritis differ in their biological features and should not be treated as the same condition. In addition, inflammatory regulation involves complex interactions among cytokines, prostaglandins, noncoding RNAs, extracellular vesicles, metabolism, and oxidative stress. Conventional treatments still have value in some cases, but microbiota-based approaches, especially probiotics, are emerging as promising tools to prevent and control disease while reducing reliance on antibiotics. A better understanding of host–microbiota interactions may support more precise strategies to improve postpartum uterine health and fertility in dairy cows.

## 1. Introduction

Postpartum uterine disease remains one of the most economically and reproductively significant disorders in modern dairy production, as it impairs uterine involution, delays conception, and reduces overall herd reproductive efficiency [1]. This clinical significance is partly attributable to the near-universal bacterial contamination of the uterine lumen after parturition; however, only a subset of cows fails to restore uterine homeostasis and consequently progresses to persistent uterine disease [2]. A key advance in the field, therefore, has been the establishment of standardized clinical definitions for puerperal metritis, clinical endometritis, and related postpartum uterine disorders, thereby enabling more consistent comparisons across epidemiological, mechanistic, and therapeutic studies [3]. Once these conditions were clearly defined, it became evident that postpartum uterine disease should not be regarded as a transient, low-impact local infection, as it exerts measurable detrimental effects on service rate, conception risk, and overall herd-level reproductive performance [4]. Large-scale field studies have since confirmed that these adverse reproductive effects persist in modern dairy production systems, highlighting that uterine disease remains a major impediment to effective fertility management [5]. As research in this field has advanced, it has become increasingly evident that postpartum uterine disease cannot be attributed solely to bacterial colonization. The host must simultaneously eliminate microbial contamination, repair tissue injury resulting from parturition, and restore reproductive tract function within a critical postpartum window [6]. This broader perspective has been reinforced by studies in transition dairy cows, which demonstrate that inflammation, immune function, and metabolic status are closely interconnected during the postpartum period. Uterine disease should therefore be viewed as in integral component of whole-animal physiology rather than an isolated disorder [7]. Accordingly, it is now recognized that the development of postpartum uterine disease is shaped by multiple interacting factors, including calving-related trauma, retained fetal membranes, metabolic stress, and impaired host defense capacity [8]. Contemporary herd-level studies further demonstrate that endometritis remains prevalent even when assessed using more refined diagnostic methods, confirming that postpartum uterine inflammation is highly common under commercial dairy management [9]. This persistent disease burden has promoted a transition from purely descriptive clinical frameworks toward more biologically integrated models of postpartum uterine health and dysfunction [10]. A further conceptual shift arose from the application of culture-independent microbiology, which showed that the uterine microbiome associated with disease development is characterized by reduced diversity and disturbed community structure, rather than the mere presence or absence of a single pathogen [11]. At the same time, recent syntheses of clinical and biological evidence have highlighted that clinical and subclinical endometritis are not merely grades of severity of a single uniform disorder, but rather distinct phenotypic manifestations of postpartum inflammatory diseases of the reproductive tract [12]. This broader perspective aligns with emerging research on mucosal immunity: the postpartum uterus is now recognized as an immunologically active site where both innate and adaptive immune pathways determine whether bacterial exposure resolves or progresses to chronic inflammation [13]. Taken together, these advances have shifted the field of research from identifying individual pathogens and towards investigating how the host–microbiota equilibrium is established after calving, how inflammatory homeostasis is maintained or disrupted, and why disease either persists or resolves in cows [14]. In this context, the present review examines bovine endometritis through the integrated lenses of clinical phenotypes, uterine microbial ecology, host immune responses, and mechanisms of inflammatory persistence, with a particular focus on how host–microbiota crosstalk regulates postpartum uterine health and disease progression.

## 2. Methodology

This article was prepared as a structured narrative review rather than a systematic review or meta-analysis. Relevant literature was identified using PubMed, Web of Science, Scopus, and Google Scholar, together with backward and forward citation tracking of key papers. The literature search covered publications from 2002 to 2025 and was designed to capture studies related to bovine postpartum uterine disease, diagnostic phenotypes, uterine microbiota, host–microbiota interactions, inflammatory regulation, metabolic status, oxidative stress, and treatment strategies in dairy cows. Search terms included combinations of “bovine”, “dairy cow”, “uterus”, “endometritis”, “clinical endometritis”, “subclinical endometritis”, “metritis”, “purulent vaginal discharge”, “uterine microbiota”, “uterine microbiome”, “dysbiosis”, “host–microbiota interaction”, “innate immunity”, “cytokine”, “prostaglandin”, “inflammatory regulation”, “non-coding RNA”, “extracellular vesicle”, “metabolism”, “NEFA”, “BHB”, “oxidative stress”, “antimicrobial treatment”, “prostaglandin F2α”, and “probiotics”. The same publication could be retrieved from more than one database; therefore, database overlap was considered during study selection, and duplicate records were removed before final inclusion. The included references were mainly indexed in PubMed, Web of Science, Scopus, and Google Scholar, with Google Scholar used as an additional broad search tool to identify relevant articles not easily captured by database-specific searches. Peer-reviewed original research articles, field studies, experimental studies, omics-based studies, clinical trials, meta-analyses, and authoritative reviews were considered eligible when they directly addressed postpartum uterine disease, diagnostic criteria, uterine microbial ecology, host–bacterial interactions, inflammatory regulation, metabolic status, oxidative stress, or treatment strategies in cattle. Studies were excluded if they were unrelated to bovine reproductive disease, focused only on non-bovine species without clear relevance to cattle, were available only as conference abstracts, lacked accessible full text, duplicated data without additional interpretation, or did not contribute directly to the conceptual framework of this review. The selection process involved screening titles and abstracts, assessing potentially relevant full-text articles, removing duplicate or irrelevant records, and identifying additional key publications through citation tracking. Because the aim was to provide an integrated conceptual synthesis rather than quantitative evidence pooling, formal meta-analysis was not performed. Formal risk-of-bias assessment was also not undertaken; however, the relevance and quality of included studies were considered narratively according to disease definition, diagnostic criteria, sampling method, study design, methodological validity, and contribution to understanding bovine endometritis. Information was extracted on study type, animal population, postpartum timing, diagnostic approach, sampling method, microbial or inflammatory variables, metabolic or therapeutic factors, and major findings. The evidence was then synthesized thematically to integrate clinical, microbiological, immunological, transcriptomic, metabolomic, and therapeutic findings into a conceptual framework of bovine endometritis as a multifactorial postpartum disorder involving disrupted uterine microbial succession, host immune activation, metabolic maladaptation, persistent inflammation, tissue injury, and impaired restoration of uterine homeostasis. The final synthesis included 112 cited publications.

## 3. Bovine Endometritis

**Figure 1:**
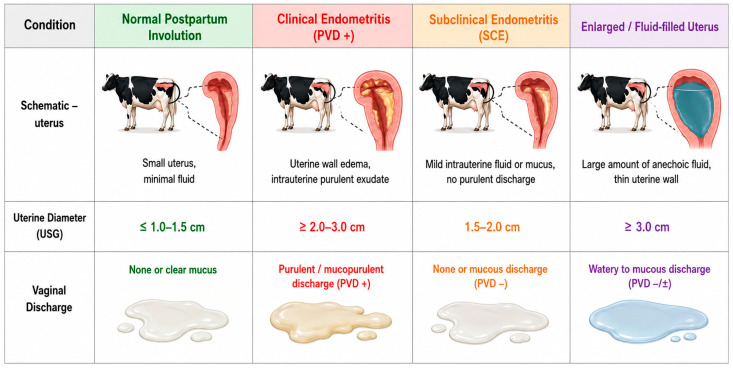
Schematic overview of diagnostic phenotypes and uterine diameter changes in postpartum bovine uterine disease.

Bovine endometritis is inflammation of the endometrium during the postpartum period. Clinical endometritis (CE) is usually diagnosed after the acute puerperal phase, typically from approximately 21 days postpartum onward, and is characterized by purulent or mucopurulent vaginal discharge without overt systemic illness [4]. Subclinical endometritis (SCE) is diagnosed cytologically by an increased proportion of polymorphonuclear neutrophils in endometrial samples, commonly using thresholds of approximately 5–10% depending on postpartum timing, sampling method, parity, and the diagnostic objective, and it occurs in the absence of visible abnormal vaginal discharge [15,16]. Therefore, postpartum uterine disease should not be reduced to a single clinical entity: cytological endometritis, clinical endometritis, and purulent vaginal discharge (PVD) represent related but non-interchangeable manifestations of reproductive-tract inflammation. In clinically normal postpartum cows, Kasimanickam et al. [15] demonstrated that endometrial cytology and ultrasonography could identify subclinical endometritis even in the absence of overt abnormal discharge. A direct comparison of diagnostic approaches by Barlund et al. [17] further showed that endometrial cytology performed using a double-guarded cytobrush was the most reliable among several commonly used field methods for diagnosing postpartum endometritis. Importantly, Westermann et al. [18] showed that vaginoscopic diagnosis of clinical endometritis may yield false-positive findings when compared with uterine bacteriology and cytology—a finding that helps explain why visible discharge does not always correlate precisely with endometrial inflammation. This diagnostic complexity was quantified by Dubuc et al. [19], who showed that different postpartum endometritis criteria identify distinct subsets of cows rather than a single, stable disease population. Building on these findings, Denis-Robichaud et al. [20] established optimized criteria for both PVD and cytological endometritis and demonstrated that each was associated with reduced odds of pregnancy at first service. Methodological nuance is also of importance, as Van Schyndel et al. [21] reported that cytobrush and low-volume lavage do not yield identical cytological results, even when both are used to assess the same postpartum uterus. More recently, Druker et al. [22] demonstrated that primiparous and multiparous cows may require different PMN thresholds and sampling times for an optimal diagnosis of cytological endometritis. However, the diagnostic accuracy of SCE remains an important methodological issue. Although endometrial cytology is widely used, SCE should not be interpreted as a completely uniform inflammatory condition across the whole endometrium. Evidence obtained from different endometrial regions indicates that inflammation may be unevenly distributed within the uterus and that active and chronic inflammatory alterations are not always spatially identical [23]. Therefore, the site of sampling can influence PMN percentages and disease classification. Studies using the cytobrush technique have further shown that PMN counts and diagnostic agreement may vary among predefined endometrial sites, suggesting that a single cytobrush sample may not fully represent the inflammatory status of the entire endometrium in all cows [24]. In addition, endometrial mRNA expression of inflammation-related mediators differs among the uterine corpus, horn bases, and horn tips, indicating that cytokine- or transcript-based assessments of uterine inflammation also require careful standardization of sampling location [25]. These findings do not invalidate cytology-based SCE diagnosis, but they indicate that SCE should be interpreted as a probabilistic estimate of endometrial inflammatory burden rather than as a perfectly fixed disease label. They emphasized that disease definition should be based on the biological context rather than on a single, universal cutoff point. The reproductive significance of this distinction becomes particularly evident when PVD is considered as an independent outcome. In a widely cited clinical field study, Runciman et al. [26] showed that cows diagnosed as positive for purulent or mucopurulent discharge by either Metricheck or vaginoscopy had exhibited poorer reproductive performance than cows with no abnormal discharge. Therapeutic studies have further reinforced the clinical relevance of PVD, as McDougall et al. [27] tested treatment protocols for cows with purulent vaginal discharge and demonstrated that management decisions regarding PVD cannot be dissociated from subsequent reproductive performance. In grazing dairy cows, Giuliodori et al. [28] reported that higher vaginal discharge scores in grazing dairy cows were associated with a lower probability of pregnancy and a longer calving-to-pregnancy interval. Ryan et al. [29] further found that PVD diagnosed at 21 days postpartum in spring-calving, pasture-based herds was influenced by previous lactation milk yield and was associated with diminished fertility. More recent evidence suggests that discharge scoring may also capture deeper biological differences. For example, Cañadas et al. [30] linked postpartum vaginal discharge scores with fertility phenotypes, metabolic status, and overall reproductive performance in pasture-based cows with seasonal calving. Building on this research, Figueiredo et al. [31] demonstrated that characteristics of vaginal discharge and fever within the first two weeks after calving were associated with subsequent productive and reproductive outcomes. Taken together, these studies suggest that the most clinically meaningful approach is to consider PVD and endometritis within a unified postpartum inflammatory continuum, rather than conflating the two conditions. In this continuum, visible vaginal discharge, endometrial cytology, ultrasonographic uterine alterations, and fertility outcomes represent related yet non-interchangeable dimensions of disease. The impact of endometritis on reproduction is now beyond dispute, as both clinical and subclinical forms are consistently associated with delayed conception and impaired fertility. In dairy cows that are repeatedly bred, subclinical endometritis has been shown to be highly prevalent and to have a measurable detrimental effect on fertility, even when there are no obvious clinical signs [32]. At the herd level, postpartum uterine diseases reduce reproductive performance and are also associated with reduced milk production, indicating that their impact extends beyond the uterus itself [33]. Metabolic status should therefore be considered an important component of postpartum uterine health, because elevated prepartum non-esterified fatty acids (NEFA), postpartum hyperketonemia, low body condition score, and systemic inflammatory markers have been associated with metritis, purulent vaginal discharge, or cytological endometritis in dairy cows [34]. Periparturient disease complexes, including metabolic and uterine disorders, are also associated with delayed resumption of estrous cyclicity, reduced pregnancy per artificial insemination, and increased pregnancy loss, further supporting the link between systemic health and reproductive outcomes [35]. Recent reviews emphasize that inflammation, immune dysfunction, hypocalcemia, excessive lipid mobilization, and metabolic imbalance interact during the transition period and can compromise subsequent reproductive function [36]. However, NEFA, BHB, and calcium should not be interpreted simply as isolated causal factors, because immune activation itself may contribute to hypophagia, lipid mobilization, increased ketone production, and altered calcium dynamics during the periparturient period. Therefore, field monitoring of BHB and NEFA is useful not as a direct diagnostic test for endometritis, but as a practical tool for identifying cows with immunometabolic maladaptation and increased susceptibility to persistent postpartum uterine inflammation [37]. Accordingly, the main challenge in bovine endometritis is no longer whether the disorder is clinically significant but how to classify it in a way that distinguishes biologically distinct disease states while maintaining diagnostic accuracy in real-world settings [38]. The diagnostic phenotypes and uterine diameter changes are summarized in Figure 1.

## 4. Uterine Microbiota in Healthy and Diseased Cows

Historically, reproductive research has generally presumed the healthy bovine uterus to be sterile, with contamination only occurring at calving [2]. However, studies based on DNA sequencing have identified bacterial signatures in the uteruses of both virgin heifers and pregnant cows. This implies that, under certain physiological conditions, the bovine uterus may act as a low-biomass microbial niche [39]. These findings do not confirm the existence of a stable, dense resident microbiome comparable to that of the gastrointestinal tract. However, they demonstrate that the sterile–non-sterile dichotomy is too simplistic to characterize uterine biology [11]. Nevertheless, this interpretation must be approached with caution, as the uterus is a low-biomass environment where contamination can profoundly influence sequencing outcomes [40]. Consequently, the key scientific issue is no longer the simple detection of bacteria in the uterus, but rather the differentiation of consistent, biologically relevant uterine microbial states from methodological noise and transient colonization [41]. The postpartum uterus is a highly dynamic ecological niche characterized by the presence of blood, lochia, and tissue debris, as well as a temporarily compromised epithelial barrier during uterine involution [6]. These conditions create a temporary environment characterized by rapid microbial turnover rather than supporting a stable bacterial community in the postpartum uterus. Research by Jeon et al. [42] demonstrated that the uterine microbiota is established by the time of calving and undergoes rapid shifts in the days immediately afterwards. Divergence between healthy and metritic cows occurs during the early stage of ecological succession rather than being fully established at birth. Miranda-CasoLuengo et al. [43] further demonstrated that cows developing postpartum endometritis exhibit delayed differentiation between vaginal and uterine microbial communities. This suggests that re-establishing reproductive tract compartmentalization is an essential aspect of uterine health. Therefore, the healthy postpartum uterus cannot be defined as bacteria-free; rather, it is characterized by coordinated microbial succession, anatomical separation, and host recovery [44]. Healthy postpartum cows typically exhibit greater diversity within their uterine microbial communities and less extreme dominance by inflammation-associated taxa than diseased individuals [45,46]. By contrast, metritis and clinical endometritis are consistently associated with reduced microbial diversity and the enrichment of taxa including *Fusobacterium*, *Bacteroides*, *Porphyromonas*, and *Trueperella*, *Escherichia*, and *Peptoniphilus*, among others [47,48,49]. These findings support a dysbiosis-driven model, whereby uterine disease arises from an ecological imbalance and pathogen-dominated microbial community structure, rather than the mere presence of a single bacterial species [44,45]. Accordingly, modern characterizations of the microbiota associated with uterine disease should emphasize microbial communities, interspecies interactions, and functional potential, rather than merely listing individual organisms [48,50]. CE and SCE are not microbiologically equivalent. Wang et al. [49] reported that CE was characterized by elevated levels of *Fusobacterium* and the presence of taxa including *Trueperella* and *Peptoniphilus.* In contrast, SCE exhibited a distinct uterine microbial profile without comparable dominance of classical pathogens. Pascottini et al. [51] further demonstrated that the uterine microbiota of cows with SCE more closely resembled that of healthy animals than that of cows with CE. CE was distinguished by a marked reduction in microbial diversity. Recent shotgun metagenomic studies support this distinction, indicating that clinical endometritis (CE) is clearly associated with pathogenic bacteria, whereas such a consistent association has not been established for subclinical endometritis (SCE) [50].This suggests that subclinical endometritis (SCE) should be considered a state of host–microbiome dysregulation rather than a simple pathogen-dominated uterine infection [12,50]. Future efforts to advance uterine microbiota research beyond descriptive ecological characterization toward mechanistic elucidation necessitate longitudinal experimental frameworks, strict contamination prevention strategies, and refined strain-level and function-oriented analytical pipelines; to achieve high-resolution microbial classification and establish causal linkages between microbiota configuration, functional genes, metabolites and inflammatory phenotypes, subsequent research ought to systematically combine multiple analytical platforms including 16S rRNA gene sequencing, NGS-driven shotgun metagenomics, metatranscriptomics, culture-aided sequencing and quantitative PCR alongside metabolomic profiling [48,50,52].

## 5. Host–Uterine Bacteria Interactions

**Figure 2:**
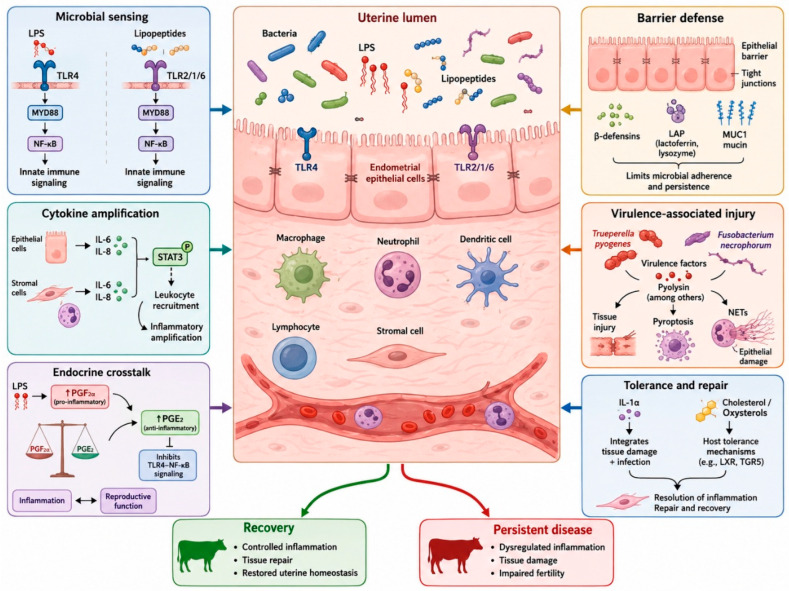
Integrated model of host–uterine microbiota interactions in postpartum bovine endometritis. The schematic illustrates how microbial ligands and virulence factors are sensed by bovine endometrial epithelial and stromal cells through TLR-dependent pathways, leading to cytokine amplification, endocrine crosstalk, epithelial barrier defense, tissue injury, and tolerance–repair responses that collectively determine whether postpartum bacterial exposure resolves into uterine recovery or progresses to persistent endometrial disease.

Interactions between host and the uterine microbiota in dairy cows should be regarded as a dynamic, bidirectional process, rather than a simplistic unidirectional model of bacterial invasion [53,54]. During the postpartum period, the endometrium is exposed to microbial ligands originating from the uterine microbiota, as well as to tissue damage, lochia release, and epithelial disruption associated with parturition. Consequently, the biological outcome depends on how microbial signals are integrated within a damaged and rapidly remodeling mucosal environment. In this context, bovine endometrial epithelial and stromal cells act as immune sentinels on the front line, rather than as passive targets. This is because they constitutively express pattern-recognition receptors (PRRs) that can detect bacterial lipopolysaccharide (LPS) and initiate innate immune defense programs [55]. This early recognition event critically depends on the TLR4–MYD88 signaling axis, which connects Gram-negative bacterial products to the downstream transcriptional activation of pro-inflammatory mediators in bovine endometrial cells [56]. Host recognition extends far beyond LPS alone; bovine endometrial epithelial and stromal cells also respond to bacterial lipopeptides via TLR2/1/6-dependent pathways. This demonstrates that the host can distinguish multiple classes of microbial molecules within the postpartum uterine environment [57]. Accordingly, host–microbiota interactions are shaped not only by the presence of bacteria, but also by the genetic and functional composition of the uterine microbial community. This determines the total amount of inflammatory ligands and virulence-associated signals to which the endometrium is exposed [46,48]. Once microbial recognition is triggered, the host response quickly moves from sensing the ligand to orchestrating inflammatory responses. Polarized bovine endometrial epithelial cells secrete IL-6 preferentially towards the apical surface, implying that uterine mucosal immunity is spatially organized and targeted at the luminal microbial interface rather than being diffusely distributed within the tissue [58]. This initial response can be sustained via IL-6 receptor/STAT3-dependent positive feedback, which prolongs IL-8 secretion and thereby amplifies leukocyte recruitment and local inflammatory tone [59]. Concurrently, bacterial stimulation modulates the endocrine function of the endometrium. For example, lipopolysaccharide redirects prostaglandin synthesis from PGF2α towards PGE2, thereby linking microbial recognition directly to luteolytic signaling and subsequent reproductive resilience [60]. Importantly, this interaction is not exclusively pro-inflammatory: PGE2 can also attenuate LPS-induced inflammatory responses via the TLR4–NF-κB pathway in bovine endometrial epithelial cells, revealing that host–microbiota crosstalk encompasses both inflammatory amplification and local counter-regulatory mechanisms [61]. A second key layer of host–microbiota interaction involves barrier defense and selective microbial responsiveness. The bovine endometrium expresses antimicrobial peptides including β-defensins and lingual antimicrobial peptide. This demonstrates that the host relies not only on inflammation driven by leukocytes, but also deploys intrinsic epithelial defense systems to modulate microbial persistence at the mucosal surface [62]. These antimicrobial programs are inherently dynamic, as the expression of distinct antimicrobial peptides varies with the physiological and inflammatory status of the endometrium [63]. Futhermore, not all uterine bacteria interact equivalently with the host: bovine endometrial epithelial cells tailor their pro-inflammatory responses according to the pathogenic potential of distinct *Trueperella pyogenes* strains. This demonstrates that the host responds not only to microbial taxonomy, but also to variation in virulence that is functionally relevant [64]. This aligns with population-level evidence indicating that the virulence factors of *Escherichia coli*, *Fusobacterium necrophorum*, and *Trueperella pyogenes* are strongly associated with postpartum uterine disease in dairy cows [65]. Furthermore, experimental infusion studies reveal that virulent *T. pyogenes* can modify endometrial inflammatory gene expression and disrupt luteal dynamics. This indicates that host–microbiota interactions must be conceptualized as both immunological and reproductive processes [66]. At the cellular level, this interaction has even greater functional significance, as bovine endometrial stromal cells are highly sensitive to pyolysin—the cholesterol-dependent cytolysin secreted by *T. pyogenes*—thereby linking bacterial virulence directly to stromal damage and compromised tissue integrity [67]. The ultimate factor in determining the outcome of a disease lies in the host’s ability to contain tissue injury while eliminating invading microbes. Rather than mounting a generic response to damage, endometrial cells utilize IL-1α as a key signal that modulates the magnitude of inflammation in response to concurrent microbial challenge and cellular injury. This process integrates pathogen detection with the severity of tissue disruption [68]. This is important because severe postpartum uterine disease is not solely driven by failed immune resistance; host tolerance mechanisms also play a significant role. Furthermore, cell-intrinsic pathways that modulate membrane cholesterol metabolism can enhance stromal cell tolerance to pyolysin-induced damage [69]. Similarly, oxysterols protect bovine endometrial cells against injury caused by pore-forming toxins, which reinforces the notion that disease severity depends on the balance between microbial virulence and host tissue resilience [70]. Recent studies have shown that LPS can trigger pyroptosis in bovine endometrial epithelial cells, suggesting that inflammatory cell death is an essential component of the pathological interaction between the host and uterine microbiota [71]. Furthermore, neutrophil extracellular traps can promote endometrial epithelial pyroptosis during endometritis, demonstrating that host defence mechanisms intended to control infection may also exacerbate epithelial damage and perpetuate chronic inflammation [72]. Taken together, host–uterine microbiota interactions in dairy cows are best conceptualized as a multistage process. In this process, the composition of the microbial community, the presence of pathogen-associated molecular patterns, toxin production, epithelial defense, stromal tolerance, and inflammatory resolution all determine whether postpartum bacterial exposure results in physiological recovery or progresses to persistent endometrial disease [54]. Systemic biochemical evidence supports this conclusion: cows with endometritis exhibit altered metabolic profiles in their serum that are associated with negative energy balance, bacterial proliferation, and immune activation. This suggests that uterine inflammation forms part of the wider postpartum systemic physiology rather than representing a strictly local uterine event. These multistage host–microbiota interactions are summarized in Figure 2.

## 6. Uterine Microbiota and Inflammatory Regulation

**Figure 3:**
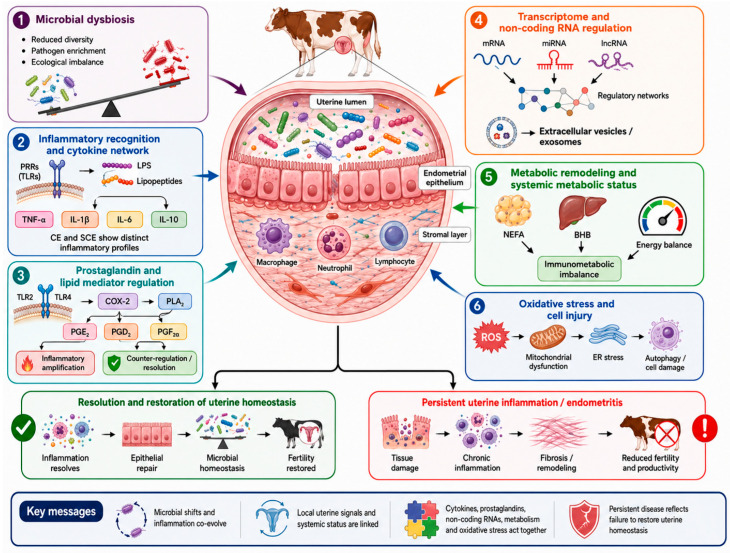
Integrated regulatory network linking uterine microbial dysbiosis to persistent inflammation and impaired postpartum uterine homeostasis in dairy cows.

The regulation of inflammation in bovine endometritis is more accurately conceptualized as a dynamic process in which shifts in the microbial community and uterine biochemical remodeling evolve together, rather than merely a passive downstream consequence of pathogen exposure, as demonstrated by the integrated metagenomic–metabolomic analyses of diseased cattle conducted by Cao et al. [52]. This systems-level perspective is further supported by evidence showing that metritis and its associated uterine disease microbiome leave enduring transcriptional signatures in the caruncular endometrium, indicating that inflammatory dysregulation persists beyond the clinical acute phase [73]. Moreover, longitudinal postpartum studies have demonstrated that the uterine inflammatory state one month after calving continues to reflect prior metritis, purulent discharge, antibiotic exposure, and estrous cyclicity, strongly arguing against the view of uterine inflammation as a transient event [74]. In the same vein, microbiome profiling of cows receiving metritis treatment has revealed that variations in uterine microbial composition are associated not only with clinical resolution but also with pregnancy rate at first insemination, thereby directly linking inflammatory recovery to reproductive performance [75]. Further support comes from metabolomic evidence: cows that fail to achieve clinical cure following metritis exhibit distinct uterine and serum metabolic profiles enriched in inflammatory and energy-related pathways, suggesting that non-resolving inflammation is metabolically entrenched rather than merely a histological finding [76]. Experimental infection studies further indicate that the consequences of uterine inflammation are not spatially confined to the endometrium. Infection-induced transcriptomic alterations remain detectable in the oviduct and granulosa cells long after the initial insult [77]. Consistent with this finding, previous uterine infection has been shown to alter the endometrial transcriptomic response to pregnancy, suggesting that inflammatory programming can leave a lasting imprint on reproductive physiology even after overt clinical disease has resolved [78]. Systemic biochemical evidence also supports this conclusion: cows with endometritis exhibit altered serum metabolic profiles associated with negative energy balance, bacterial proliferation, and immune activation. This indicates that uterine inflammation is integrated into the broader systemic postpartum physiology [79]. Transcriptomic analyses at the local tissue level reveal that inflammatory regulation in the bovine endometrium encompasses far more than just a restricted cytokine response. Global profiling of lipopolysaccharide-exposed bovine endometrial cells has revealed the activation of interferon-responsive genes, immunoproteasome pathways, and intracellular pathogen-recognition programs, which together demonstrate that a bacterial challenge can elicit extensive immune reprogramming [80]. Whole-transcriptome analysis of bovine endometrial epithelial cells also supported the concept that lipopolysaccharide profoundly remodels the expression of genes related to innate immune and inflammation, and that epithelial cells play a key role in actively governing the uterine inflammatory set point [81]. Parallel RNA-seq analyses of bovine endometrial stromal cells similarly revealed similar comprehensive responses to lipopolysaccharide, underscoring the tissue-specific nature of inflammatory regulation within the endometrium [82]. Functional stimulation experiments further demonstrated that bovine endometrial cells respond not only to lipopolysaccharide, but also to IL-1β and TNF-α. This indicates that multiple inflammatory signals converge within the same tissue to amplify or reshape uterine immune responses [83]. At the same time, inflammatory regulation is not unilaterally pro-inflammatory. For example, prostaglandin E2 can inhibit LPS-induced responses via the TLR4–NF-κB pathway in bovine endometrial epithelial cells, revealing the existence of local counter-regulatory mechanisms [61]. However, lipid mediators can also exacerbate pathology. Toll-like receptors 2 and 4 stimulate prostaglandin E_2_ synthesis in *Escherichia coli*–infected bovine endometrial tissue, thereby exacerbating tissue injury [84]. More recent mechanistic studies have refined this concept, demonstrating that the TLR2/TLR4/NLRP3–H-PGDS–PGD2 axis exerts dual regulatory roles in *Escherichia coli*–induced endometritis. This highlights that prostaglandin signaling acts as a central regulatory hub rather than merely as a downstream effector [85]. Clinical sampling studies align with these mechanistic findings: concentrations of TNF-α, IL-1β, IL-6, and IL-10 in uterine lavage fluid and serum differ significantly between healthy cows, cows with subclinical endometritis, and cows with clinical endometritis. This confirms that local and systemic inflammatory compartments remain biologically interconnected [86]. A related cytokine study further indicated that subclinical endometritis may be characterized by an IL-10-dominated microenvironment, which impairs local uterine defense, promotes microbial persistence, and hinders effective bacterial clearance [87]. This biological distinction is further supported by whole-transcriptome comparisons between clinical and subclinical endometritis, which identified distinct transcriptomic and miRNomic endometrial signatures underlying the two phenotypes [88]. Cell-type-resolved analyses reinforce this conclusion further: subclinical endometritis differentially modifies transcriptional programs in the luminal, glandular, and stromal compartments, suggesting that inflammatory persistence is spatially partitioned within the endometrium [89]. Another significant development in this area is the realization that inflammatory regulation is influenced not only by conventional cytokines and prostaglandins, but also by non-coding RNAs and extracellular vesicle signaling. Circulating cell-free microRNA profiles differ in cows with persistent subclinical endometritis, indicating that uterine inflammation is reflected in a detectable systemic regulatory RNA signature [90]. Network-based systems analysis integrating mRNAs, lncRNAs, and miRNAs has also identified co-expression modules and hub regulators implicated in bovine endometritis. This demonstrates that inflammatory regulation relies on multilayered transcriptomic crosstalk rather than individual differentially expressed genes [91]. Recent integrative analyses of metritis-associated non-coding RNA networks have reached the same conclusion, demonstrating that differentially expressed miRNAs and lncRNAs are functionally embedded within core inflammatory pathways underlying postpartum uterine disease [92]. Functional studies indicate that these RNAs serve a purpose beyond merely being descriptive biomarkers. For example, exosome-derived uterine miR-218 has been identified as both a biomarker and a mechanistic regulator of bovine endometritis via TGIF2/TGF-β–related signaling [93]. Similarly, miR-223 has been shown to inhibit NLRP3 inflammasome activation and thus play a protective role in bovine endometritis, providing a direct link between microRNA function and the resolution of inflammation [94]. Extracellular vesicles add an additional layer of intercellular regulation. For example, plasma exosomes from cows with a uterine infection decrease PGF2α synthesis in bovine endometrial epithelial cells, thereby modulating inflammatory–endocrine crosstalk within the uterus [95]. Furthermore, small extracellular vesicles from cows with high and low fertility elicit distinct expression profiles of inflammatory mediators and prostaglandin-synthesis genes in bovine endometrial cells. This implies that signals mediated by extracellular vesicles may modulate uterine inflammatory capacity prior to the onset of overt disease [96]. Metabolic and stress-response pathways are also key regulators of uterine inflammation. Persistent uterine inflammation correlates with elevated circulating levels of adiponectin, TNF-α, IL-1β, and IL-6, suggesting that non-resolving endometritis is associated with systemic endocrine–immune dyshomeostasis [97]. Metabolomic profiling of uterine secretions has revealed that cows with endometritis display distinct small-molecule signatures compared with healthy individuals, supporting the view that the inflammatory uterine microenvironment undergoes extensive metabolic remodeling alongside microbial dysbiosis [98]. Prospective metabolomic profiling of vaginal discharge has similarly identified metabolic signatures associated with the development and resolution of metritis, suggesting that inflammatory regulation can be monitored using accessible biochemical markers throughout disease progression [99]. Redox biology adds an additional mechanistic layer: oxidative stress injures bovine endometrial epithelial cells via mitochondria-dependent pathways, implying that inflammatory regulation is governed not only by canonical immune signaling but also by mitochondrial integrity [100]. A follow-up study further demonstrated that reactive oxygen species induce damage to bovine endometrial epithelial cells via the cytochrome c–mPTP pathway, reinforcing the link between oxidative injury and epithelial dysfunction in an inflamed uterus [101]. Autophagy-related mechanisms are additionally implicated, as *Escherichia coli*–induced inflammatory injury in bovine endometrial epithelial cells is mediated by coordinated disruptions in calcium mobilization, mitochondrial dysfunction, and endoplasmic reticulum stress [102]. Taken together, these studies illustrate that the crosstalk between the uterine microbiota and inflammatory response in dairy cows is governed by a multilayered regulatory network integrating microbial ecology, lipid mediators, cytokine signaling, noncoding RNAs, extracellular vesicles, metabolic status, and organelle stress. Persistent uterine disease arises when these interconnected regulatory layers fail to re-establish postpartum uterine homeostasis [97]. The integrated regulatory network is summarized in Figure 3.

## 7. Treatment of Endometritis

Treatment of bovine endometritis should be matched to the disease phenotype, postpartum timing, reproductive objective, and antimicrobial-stewardship context. For clinical endometritis or purulent vaginal discharge, intrauterine cephapirin has been supported by field trials; however, because therapeutic responses in the LeBlanc et al. trial depended on diagnostic category, cow status, and the reproductive outcome evaluated, cephapirin should be viewed as useful in selected clinical cases rather than as a general recommendation for antimicrobial treatment [103]. Its use should therefore be balanced against antimicrobial-stewardship principles, withdrawal requirements, herd policy, and fertility goals. PGF2α should also be interpreted cautiously, because an updated meta-analysis found no consistent overall improvement in calving-to-first-service or calving-to-conception intervals after PGF2α treatment for bovine endometritis [104]. Supportive transition-cow management remains important, including prevention of retained fetal membranes and calving trauma, monitoring of body condition and metabolic risk, evaluation of ovarian status, and early identification of cows with persistent postpartum inflammation. There is a stronger rationale for emphasizing probiotics, as postpartum uterine disease is now understood in terms of the body’s ability to avoid, tolerate, and resist pathogenic bacteria, rather than simply the presence of bacterial. This makes microbiota-oriented interventions a biologically coherent adjunct to conventional therapy [14]. This interpretation is reinforced by a recent review showing that dysbiosis and biofilm formation contribute to uterine disease and treatment failure, whereas probiotics are regarded as a promising tool for preventing uterine disease and restoring a healthier reproductive microbial ecosystem [44]. Initial mechanistic studies identified the therapeutic potential of selected lactic acid bacteria, demonstrating that four candidate strains could modulate *Escherichia coli* infection and inflammation in bovine endometrial cells, thereby providing a rational basis for strain selection prior to in vivo use [105]. Subsequent work using a defined three-strain consortium demonstrated marked reductions in E. coli infection, as well as the suppression of CXCL8, IL1B, IL-8, IL-1β, and IL-6, in bovine endometrial models. This provides direct proof that probiotics can lower both the burden of pathogens and the inflammatory response simultaneously [106]. In vivo, this probiotic effect appears to be mainly mediated through ecological modulation of the caudal reproductive tract rather than simple endometrial colonization. This is because intravaginal lactic acid bacteria reduced E. coli abundance and lowered uterine infection markers such as β-defensins and MUC1, despite the administered strains not being recovered from the endometrium itself [107]. The periparturient intravaginal administration of lactic acid bacteria also accelerated uterine involution. Under the more intensive schedule, it was associated with an earlier resumption of ovarian cyclicity. This is clinically relevant because faster restoration of uterine and ovarian function should reduce the period during which postpartum inflammation can persist [108]. Earlier herd-level evidence further showed that intravaginal lactic acid bacteria reduced the occurrence of purulent vaginal discharge and lowered plasma haptoglobin concentrations after calving, indicating that the benefits of probiotics may extend beyond local microbial competition to the attenuation of the systemic acute-phase response [109]. A randomized pre-calving study then showed that repeated intravaginal dosing reduced the prevalence of metritis by up to 58% compared with untreated controls. However, postpartum intrauterine administration was not superior, highlighting the fact that the timing and route of delivery are major determinants of efficacy [110]. In cows with subclinical endometritis, intrauterine administration of *Lactobacillus buchneri* DSM 32407 was associated with a more favorable uterine environment and improved reproductive performance; however, this finding should be interpreted cautiously because reproductive performance also improved in healthy cows, and no clear difference in clinical uterine status was detected between treated and placebo cows [111]. Therefore, *L. buchneri* should be presented as a promising but still context-dependent adjunctive intervention rather than as an established treatment for SCE. However, the largest field trial to date found that prepartum intravaginal probiotics did not uniformly reduce metritis across all farms, and that the clearest fertility benefits were mainly observed in multiparous cows. Therefore, probiotics are best presented as an evidence-based but management-dependent adjunctive strategy rather than a universal replacement for conventional therapy [112].

## 8. Conclusions

Bovine endometritis is a multifactorial postpartum disorder caused by failure to restore uterine homeostasis after calving. Its development reflects disrupted microbial succession, epithelial and stromal immune activation, metabolic maladaptation, tissue injury, and defective inflammatory resolution. Clinical endometritis, SCE, and PVD should be considered related but biologically and diagnostically distinct manifestations of postpartum reproductive-tract inflammation. Accurate diagnosis requires attention to sampling method, sampling site, postpartum timing, and outcome-specific thresholds. Conventional therapies remain useful in selected cases, whereas probiotics and other microbiota-oriented strategies are promising adjuncts whose efficacy depends on strain, route, timing, parity, and farm context. Future studies should be longitudinal, strain-resolved, function-oriented, and metabolically informed to support precise, fertility-preserving, and antimicrobial-sparing control of postpartum uterine disease.

## Data Availability

No new data were created or analyzed in this study. Data sharing is not applicable to this article.

## Abbreviations

The following abbreviations are used in this manuscript:

CE	clinical endometritis
SCE	Subclinical Endometritis
PVD	Purulent Vaginal Discharge
PMN	Polymorphonuclear Neutrophils
PRRs	Pattern-Recognition Receptors
LPS	Lipopolysaccharide
TLR	Toll-Like Receptor
MYD88	Myeloid Differentiation Primary Response 88
IL	interleukin
TNF-α	Tumor Necrosis Factor alpha
STAT3	Signal Transducer and Activator of Transcription 3
NF-κB	Nuclear Factor kappa B
PGF2α	Prostaglandin F2 alpha
PGE2	Prostaglandin E2
PGD2	Prostaglandin D2
H-PGDS	Hematopoietic Prostaglandin D Synthase
NLRP3	NOD-, LRR- and Pyrin Domain-Containing Protein 3
RNA-seq	RNA Sequencing
mRNA	messenger RNA
lncRNA	long non-coding RNA
miRNA	microRNA
TGIF2	TGFB-induced factor homeobox 2
TGF-β	transforming growth factor beta
CXCL8	C-X-C motif chemokine ligand 8
MUC1	mucin 1
mPTP	mitochondrial permeability transition pore
NGS	next-generation sequencing
qPCR	quantitative polymerase chain reaction
LAP	lingual antimicrobial peptide
NETs	neutrophil extracellular traps
LXR	liver X receptor
TGR5	Takeda G protein-coupled receptor 5
NEFA	non-esterified fatty acids
BHB	beta-hydroxybutyrate
ROS	reactive oxygen species
ER	endoplasmic reticulum
COX-2	cyclooxygenase-2
PLA2	phospholipase A2
USG	ultrasonography
